# Organoids-on-a-Chip: An Integrating New Technology for Drug Development

**DOI:** 10.3390/ijms27167432

**Published:** 2026-08-20

**Authors:** Mengqi He, Yuting Gong, Xinmei Xu, Menglin Hui, Fei Li, Yongjian Ai

**Affiliations:** 1 MOE Key Laboratory of Precision Nutrition and Food Quality, Department of Nutrition and Health, China Agricultural University, Beijing 100193, China; hemengqi@ibms.pumc.edu.cn (M.H.);; 2 State Key Laboratory of Respiratory Health and Multimorbidity, Institute of Basic Medical Sciences Chinese Academy of Medical Sciences, School of Basic Medicine, Peking Union Medical College, Beijing 100005, China; 3 MOE Key Laboratory of Bioorganic Phosphorus Chemistry and Chemical Biology, Department of Chemistry, Tsinghua University, Beijing 100084, China; 4 State Key Laboratory of Natural Medicines, China Pharmaceutical University, Nanjing 210098, China

**Keywords:** organoids-on-a-chip, microfluidic chip technology, multi-organ integration, drug development, drug assessment

## Abstract

Organoids-on-a-chip integrate the three-dimensional structural fidelity of organoids with the dynamic microenvironmental control capabilities of microfluidic technology, providing a transformative preclinical research platform for drug development. This perspective catalogues organoids-on-a-chip systems encompassing liver, heart, tumor, neural, tissue barrier, and multi-organ integration platforms, and elaborates on their distinctive advantages in dynamically simulating the complete in vivo trajectory of drug absorption, distribution, metabolism, target engagement, and toxicological response. The mechanisms of this technology overcome the limitations associated with conventional static culture models, and interspecies disparities are elucidated. Furthermore, the applications of these systems across critical stages of drug development are summarized, including preclinical safety assessment, integrated pharmacokinetic/pharmacodynamic studies, personalized therapeutics, immunotherapy evaluation, and disease molecular mechanisms. Finally, we highlighted the main challenges facing this field and discussed future directions.

## 1. Introduction

Drug research and development has long been plagued by persistently high attrition rates. Studies indicate that approximately 90% of candidate drugs fail during clinical trials, with roughly half attributed to insufficient efficacy and one-quarter to safety deficiencies. The root causes of these costly failures can often be traced to inherent limitations of traditional preclinical models [1]. Although two-dimensional cell lines are amenable to high-throughput screening, they lack three-dimensional spatial architecture, cell–cell interactions, and physiological metabolic and barrier functions. While animal models offer systemic complexity, they exhibit significant interspecies disparities in drug metabolism, immune responses, and disease mechanisms [2]. These limitations cause numerous drug candidates that perform well in animal studies to fail in human trials, constituting a fundamental bottleneck in clinical translation efficiency.

Organoids are three-dimensional constructs derived from stem cell self-assembly that faithfully recapitulate the cellular diversity, spatial architecture, and gene expression signatures of native human tissues [3]. Building upon this, organoids-on-a-chip are microfluidic platforms that maintain these self-assembled 3D structures under continuous perfusion, thereby preserving their intrinsic heterogeneity and organizational complexity. By contrast, organs-on-a-chip are microfluidic systems engineered to replicate the functional units of specific organs through the co-culture of multiple cell types and the application of mechanical stimuli, thus achieving organ-level physiological responses. At a more integrative level, microphysiological systems encompass a broader category that combines multiple organ-on-a-chip modules or other tissue constructs to emulate inter-organ communication and systemic pharmacokinetics. Organoids-on-a-chip platforms provide dynamically controllable microenvironments, encompassing regulated fluid flow, mechanical stimulation, tissue barrier simulation, and real-time sensing capabilities. Organoids-on-a-chip technology integrates the biological complexity of organoids with the engineering precision of microfluidic systems, offering a viable pathway for constructing more physiologically relevant drug testing models [4]. In 2025, organoids-on-a-chip and related organoid technologies were positioned as key enablers in the progressive transition from conventional animal testing toward new approach methodologies. Subsequently, these platforms have been explicitly recognized as strategic research priorities, with growing emphasis on innovation, validation, and practical application. These concerted efforts have collectively generated significant momentum for the development and broader adoption of organoids-on-a-chip systems [5]. However, a significant gap remains between current technical capabilities and clinical translation demands, urgently necessitating the construction of human-derived in vitro models that possess both physiological relevance and predictive power. Although numerous studies have demonstrated the structural simulation capabilities of organoids, research systematically validating their predictive capacity for human drug responses remains extremely limited [6].

**Figure 1:**
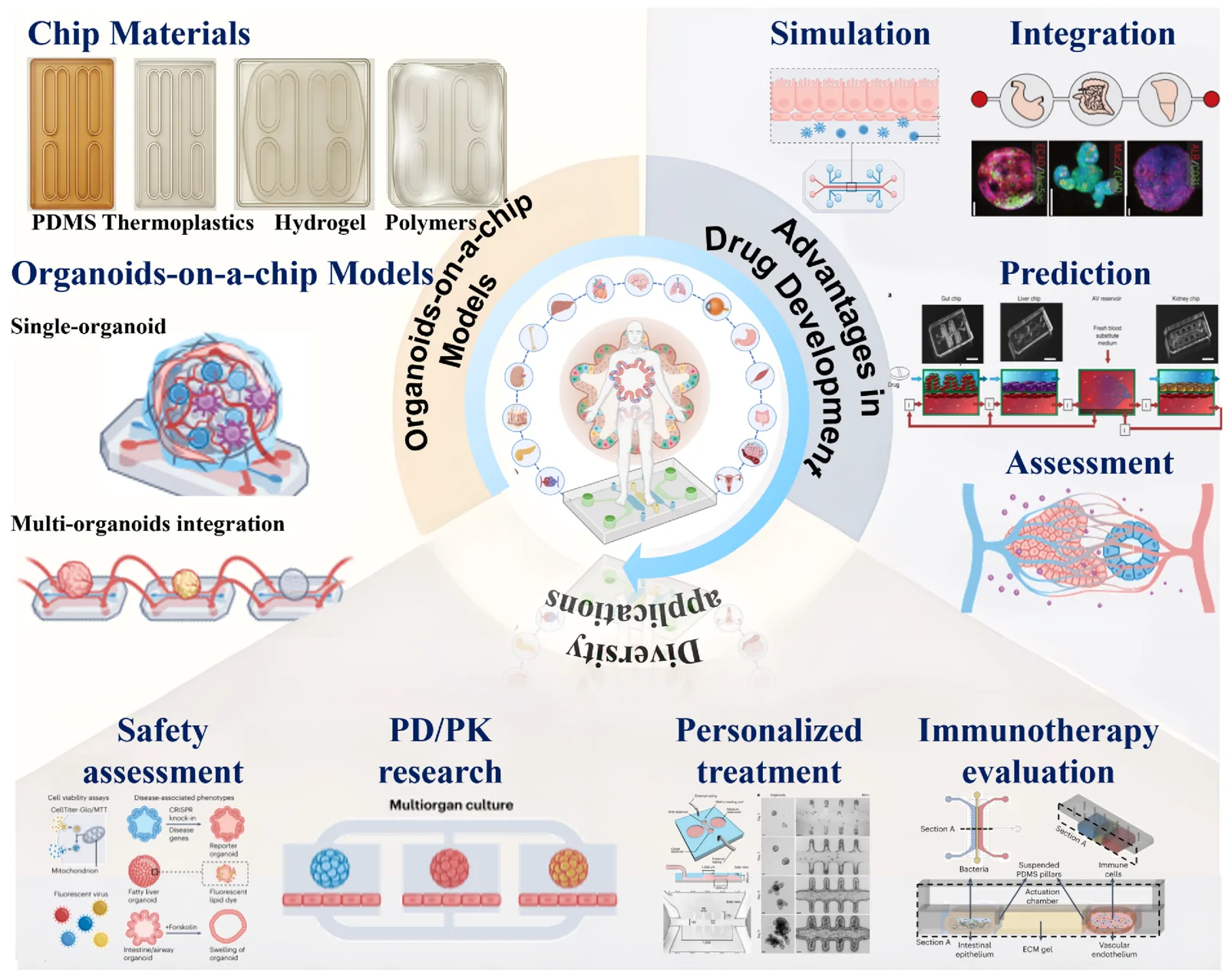
The organoids-on-a-chip as an integrating new technology for drug research and development.

This perspective advances a central thesis about how the valuation of organoids-on-a-chip must urgently shift from a paradigm of morphological similarity to functional reconstruction. Specifically, the core value of this technology lies not in its structural resemblance to human organs, but in its ability to engineer the complete sequential trajectory of drug action, from absorption, distribution, metabolism, and target engagement to toxicological response. This perspective catalogs organoids-on-a-chip model systems spanning liver, cardiac, tumor, neural, tissue barrier, and multi-organ integrated platforms; elucidates the mechanisms by which they overcome the limitations of conventional static culture models and interspecies disparities; summarizes their translational applications across critical drug development stages, including preclinical safety assessment, integrated pharmacokinetic/pharmacodynamic studies, personalized therapeutics, and immunotherapy evaluation; and discusses the core challenges and future directions confronting the field, encompassing standardization, scalability, vascularization, immune integration, and regulatory acceptance (Figure 1).

## 2. Organoids-on-a-Chip and Characteristics

Organoids-on-a-chip integrate three-dimensional organoid culture systems with microfluidic chip technology to construct dynamically controllable microphysiological environments, with their core feature being the paradigm shift from traditional static culture to dynamic microenvironmental control [7]. Specifically, the platform first preserves the inherent cellular diversity and self-organizing properties of organoids, maintaining the genotype and phenotype of original tissues with high fidelity. For example, patient-derived tumor organoids retain driver gene high mutations rates, providing a reliable genetic basis for personalized drug screening. Second, continuous perfusion enables dynamic nutrient supply and metabolic waste clearance, simulating the flow of interstitial fluid in vivo while effectively overcoming the limited nutrient gradients and metabolite accumulation characteristic of conventional static culture [8]. Third, integrated, precisely controllable physical stimulation modules apply mechanical cues such as fluid shear stress and cyclical stretch to simulate in vivo mechanical microenvironments, including cardiac pulsation, respiratory motion, and intestinal peristalsis. Thereby recapitulating the regulatory roles of mechanical factors in cellular behavior. Finally, functional interconnection of different organoid modules via microfluidic networks achieves sequential exposure and transfer of drugs and their metabolites across multiple organs, simulating systemic multi-organ collaborative metabolism and pharmacodynamic/toxicological cascade effects [9]. The synergistic integration of these features enables organoids-on-a-chip to overcome the fundamental limitations of traditional two-dimensional static culture models in physiological relevance, offering a high-fidelity in vitro testing platform that recapitulates organ-level functions and interactions for drug development.

## 3. Organoids-on-a-Chip

### 3.1. Chip Fabrication Materials

The performance of organoids-on-a-chip systems depends not only on the biological fidelity of the embedded organoids and the architectural design of microfluidic networks, but also on the physicochemical properties of the constituent fabrication materials. Material selection directly governs critical operational parameters, including optical transparency, gas permeability, biocompatibility, and mechanical properties [10]. A systematic survey of the physicochemical characteristics of available materials and their compatibility with organoid culture is therefore essential to advance the field from proof-of-concept prototypes toward standardized, reproducible platforms. This section systematically reviews the prevailing fabrication materials, addressing elastomers, thermoplastics, hydrogels, and novel functional polymers in sequence (Table 1).

Among conventional elastomeric materials, polydimethylsiloxane (PDMS) remains the most widely adopted substrate, owing to its excellent optical transparency, gas permeability, biocompatibility, and facile moldability, which also facilitate straightforward integration of mechanical actuation modules; its inherent hydrophobicity and tendency for nonspecific adsorption of small-molecule drugs can alter drug concentration profiles in pharmacokinetic studies, an important consideration in the design of drug-testing experiments [11]. Beyond these general concerns, a comprehensive assessment of PDMS performance in organ-on-a-chip systems must account for multiple interrelated factors: compound properties, including hydrophobicity, molecular weight, and logP, determine the partitioning and absorption of analytes into the PDMS matrix, with highly lipophilic molecules exhibiting greater retention; device geometry, such as channel dimensions, surface-to-volume ratio, and flow path design, critically affects mass transfer and the effective cellular exposure to test compounds; surface treatment, e.g., plasma oxidation or coating with hydrophilic polymers or proteins, can substantially reduce nonspecific binding and alter absorption kinetics; exposure time also warrants consideration, as short-term acute assays may experience minimal drug depletion, whereas long-term culture can lead to progressive sequestration of hydrophobic compounds; and finally, medium composition, particularly serum content, protein binding, and lipid presence competes with PDMS for hydrophobic drugs, thereby modifying their free concentration available to cells. Collectively, these variables dictate the magnitude of PDMS-related artifacts, and careful optimization of experimental parameters is essential to mitigate such effects. In parallel, thermoplastic polymers such as polycarbonate, polymethyl methacrylate, and cyclic olefin copolymers have attracted increasing attention [12]. These materials combine high optical clarity with low small-molecule adsorption, which minimizes drug loss at channel walls and improves quantitative reliability, thus offering performance characteristics that may be advantageous in specific contexts. Regarding biomimetic soft materials and novel functional polymers, hydrogels are widely employed as extracellular-matrix mimics because of their tunable mechanical properties and biomimetic characteristics, providing three-dimensional culture scaffolds that approximate in vivo conditions for organoids, while novel functional polymers including selenium-tellurium-based materials, thiourethane-ethers, and their methacrylate derivatives exhibit tunable mechanical properties, low small-molecule adsorption, and facile surface functionalization; compatible with diverse microfabrication processes, these materials further expand the design space for structural innovation and functional integration in organoids-on-a-chip systems.

### 3.2. Organoids-on-a-Chip Models

In recent years, organoids-on-a-chip has matured into a comprehensive model system covering a wide range of biological scenarios [13]. These diverse models enable human-relevant assessments of drug metabolism, organ-specific toxicity, anti-tumor efficacy, barrier permeability and systemic pharmacokinetics. Consequently, this subsection will provide a brief review, categorized by type, of recent research advances achieved with these organoids-on-a-chip platforms.

#### 3.2.1. Metabolic Organs

Liver organoids-on-a-chip systems represent the most advanced models in terms of regulatory acceptance, primarily employed for drug-induced liver injury risk assessment and drug metabolic stability studies [14]. Their regulatory traction stems from their ability to provide human-relevant predictions of hepatotoxicity and clearance rates, thereby reducing reliance on animal models in early-stage safety evaluations. These models typically utilize microfluidic devices to achieve multi-cellular co-culture, incorporating human hepatocyte organoids alongside endothelial cells, Kupffer cells, and stellate cells under controlled physiological flow conditions. This dynamic perfusion not only supplies continuous nutrients and oxygen but also applies shear stress, which preserves hepatic polarity and metabolic enzyme activity over extended culture periods. The inclusion of Kupffer cells enables immunotoxicology assessment, while stellate cells allow monitoring of fibrotic responses, making these platforms suitable for chronic exposure studies. Intestinal organoids-on-a-chip platforms, in parallel, are chiefly applied to the study of oral drug absorption and intestinal barrier function. These systems recapitulate villus-like structures and mucus-producing goblet cells, enabling precise measurement of paracellular and transcellular permeability, efflux transporter activity, and first-pass metabolism, thus providing critical insights into bioavailability and food–drug interactions.

#### 3.2.2. Effector/Toxicity Target Organs

The cardiac organoids-on-a-chip system combines periodic electrical stimulation with real-time recording of contractile force, thereby enabling the assessment of chronic cardiotoxicity and distinguishing between acute and delayed toxic responses. The renal organoids-on-a-chip platform is primarily used for the assessment of tubular toxicity and can be connected in series with liver, cardiac, or other modules to elucidate secondary nephrotoxicity caused by hepatic metabolism. The neural organoids-on-a-chip system encompasses the blood–brain barrier, cortical organoids, and models of neurodegenerative diseases, which are used respectively to study the mechanisms of drug penetration across the blood–brain barrier, to evaluate the activity of drugs in the central nervous system, and to screen for compounds capable of modulating the progression of neurodegenerative diseases [15].

#### 3.2.3. Tumor Systems

Tumor organoids-on-a-chip platforms reconstruct functionally active tumor microenvironments by integrating patient-derived tumor organoids with autologous immune cells or vascular endothelial components [16]. These models demonstrate distinctive advantages in personalized drug screening and immunotherapy response prediction, with colorectal cancer [17] and glioblastoma [18] organoids-on-a-chip models having shown promising correlations with immune checkpoint inhibitor efficacy and patient stratification in preliminary retrospective studies; however, prospective clinical validation remains to be established.

#### 3.2.4. Tissue Barriers

Tissue barrier organoids-on-a-chip models cover the blood–brain barrier, intestinal, skin, pulmonary, renal, and retinal barriers, predominantly adopting dual-channel designs with porous membranes or hydrogel membranes separating distinct tissue compartments [19]. Notably, emerging membrane-free hydrogel-based barrier models have achieved significant advances in mechanical microenvironment fidelity by directly mimicking basement membrane functions through extracellular matrix hydrogels, approximating the physiological states of native tissues more closely.

#### 3.2.5. Multi-Organ Integrated Systems

Multi-organ organoids-on-a-chip platforms serially integrate functional modules, including intestinal, hepatic, cardiac, renal, and neural organoid tissues via microfluidic networks, simulating the complete in vivo circulation trajectory of drug absorption, metabolism, distribution, and excretion for systemic toxicity assessment and inter-individual metabolic variability evaluation [20]. The development of modular connectivity strategies has enabled flexible assembly of organoid units, substantially lowering the technical threshold for multi-organoids integration and providing a technical foundation for constructing customizable ADME testing platforms.

## 4. Advantages of Organoids-on-a-Chip in Drug Development

Organoids-on-a-chip integrating three-dimensional human cell architectures, dynamic fluidic perfusion, physiologically relevant mechanical stimulation, and interconnected multi-organ configurations. This integrative approach systematically addresses the two fundamental limitations of conventional preclinical models: the static, closed culture conditions that fail to recapitulate circulatory dynamics and physiological gradients, and the persistent interspecies disparities that confound extrapolation of human drug responses. Fluidic perfusion not only supplies continuous nutrients and removes metabolic waste, but also applies shear stress that preserves cellular polarity, tissue-specific differentiation, and long-term functional stability. Mechanical stimulation, such as cyclic stretch or compression, mimics native organ motions like breathing or peristalsis, further promoting maturation of both parenchymal and stromal components [21]. Multi-organ connectivity enables serial drug exposure and metabolite transfer across compartmentalized tissues, simulating whole-body pharmacokinetics and revealing systemic inter-organ crosstalk features unattainable in conventional assays. By synergistically combining these engineering and biological elements, organoids-on-a-chip offer human-relevant predictive platforms for efficacy, toxicity, and absorption, thereby reducing the risk of clinical trial failures. This section delineates their distinctive advantages across four dimensions, encompassing physiological recapitulation, predictive accuracy, personalized medicine potential, and scalability for high-throughput screening.

### 4.1. Disease Heterogeneity and Microenvironment Simulation

Conventional models rely on immortalized cell lines or genetically engineered animals, failing to recapitulate patient-specific heterogeneity at genomic and microenvironmental levels. Patient-derived organoids (PDOs) retain donor mutation landscapes and pathophysiological hallmarks, improving drug response prediction. Organoids-on-a-chip platforms further integrate autologous immune cells, stromal fibroblasts, and endothelial components under dynamic perfusion, reconstructing functionally active disease microenvironments [22]. By preserving the disease–stroma–immune interaction network, these platforms provide high-fidelity systems for personalized screening, immunotherapy evaluation, and mechanistic investigation.

### 4.2. Multi-Organoids Integration and ADME Research

Single-organ models cannot recapitulate integrated absorption, distribution, metabolism, excretion (ADME) processes or evaluate inter-organ metabolite distribution [23]. Multi-organoids-on-a-chip serially interconnect intestinal, hepatic, cardiac, and renal modules via microfluidic networks, simulating complete systemic circulation from intestinal absorption through hepatic first-pass metabolism to renal excretion. This strategy permits simultaneous resolution of systemic pharmacokinetics and organ-specific pharmacodynamic or toxicological effects within a single platform, furnishing a foundation for customized pharmacokinetic–pharmacodynamic research.

### 4.3. Human-Specific Metabolism and Toxicity Prediction

Conventional screening relies on animal models or two-dimensional hepatocytes: the former introduces systematic deviations due to interspecies differences in cytochrome P450 enzymes, transporters, and toxicity pathways; the latter suffers from polarity loss and limited survival, precluding integrated metabolic function. Liver organoids-on-a-chip systems integrate hepatocytes, endothelial cells, stellate cells, and Kupffer cells under controlled perfusion, maintaining polarity, enzyme activity, and bile secretion for weeks through dynamic nutrient delivery and physiological shear forces [24]. This strategy circumvents predictive failures from both interspecies disparities and static limitations, offering human-specific assessment for drug-induced liver injury.

### 4.4. Pharmacodynamic Assessment and Functional Endpoints

Traditional pharmacodynamic evaluation depends on animal behavioral indices or static two-dimensional assays, precluding real-time, organ-level resolution of drug actions. Animal models yield systematic deviations in target validation due to species-specific receptor and pathway differences; two-dimensional systems lack functional readouts for organ-level physiology such as contractility or electrophysiological conduction [25]. Organoids-on-a-chip platforms incorporate biosensors, real-time imaging, and electrophysiological modules for dynamic, multi-parameter assessment. Cardiac organoids chips monitor contractile force and electrical propagation; neural organoids chips capture synaptic plasticity; hepatic organoids chips quantitate albumin synthesis and bile acid transport. This paradigm, centered on functional recovery rather than cell death endpoints, provides high-temporal-resolution evidence for mechanism elucidation and dosing optimization. In multi-organoids systems, pharmacodynamic assessment proceeds synchronously with ADME, correlating concentration–time profiles with functional responses to furnish comprehensive pharmacokinetic–pharmacodynamic frameworks for therapeutic window delineation.

## 5. Applications of Organoids-on-a-Chip in Drug Development

### 5.1. Preclinical Safety Assessment

Organoids-on-a-chip integrate human parenchymal cells, stromal components, and tissue-resident immune cells to reconstruct physiologically relevant target-organ microenvironments under dynamic perfusion, offering a more human-specific alternative for preclinical drug evaluation [26]. Liver-on-chip platforms simulate the injurious effects of reactive metabolites generated during drug bioactivation on hepatocytes, while enabling the dissection of immune cell-mediated inflammatory cascades. Cardiac organoids-on-a-chip identify drug-induced perturbations of myocardial ion channels and cytoskeletal integrity through simultaneous monitoring of electrophysiological signals and contractile mechanics. Renal tubule chips focus on direct damage to epithelial barrier function and transporter activity caused by drugs and their metabolites. Multi-organ serial systems further elucidate secondary toxicities of hepatically generated metabolites in distal organs such as the heart or kidney, compensating for the inability of single-organ models to assess systemic toxicity risks.

### 5.2. Pharmacokinetic–Pharmacodynamic Integration

Organoids-on-a-chip functionally serialize absorption, metabolism, distribution, and excretion modules via microfluidic networks, enabling drugs and their metabolites to sequentially expose each target organ according to physiological chronology and thereby simulating complete systemic circulation trajectories [27]. Researchers can simultaneously capture dynamic drug concentration profiles and organ functional indices within a single platform, establishing direct quantitative exposure–effect relationships. The modular architecture permits flexible configuration of organ combinations, connection sequences, and fluidic parameters based on the physicochemical properties, metabolic pathways, and target-organ distribution characteristics of candidate drugs, enabling customized simulation of diverse dosing scenarios and providing a high-temporal-resolution experimental foundation for integrated pharmacokinetic–pharmacodynamic research.

### 5.3. Personalized Medicine and Companion Diagnostics

Patient-derived organoids-on-a-chip platforms retain donor-specific genetic mutation landscapes, epigenetic signatures, and microenvironmental components, furnishing highly individualized in vitro systems for drug sensitivity assessment [28]. Compared with the prolonged engraftment and observation periods required by conventional patient-derived xenograft models, organoids-on-a-chip enable parallel multi-regimen screening and rapid response evaluation, substantially compressing the temporal window from specimen acquisition to clinical decision-making. These platforms systematically evaluate the inhibitory effects of chemotherapeutic, targeted, and combination regimens on individual tumor tissues, while dissecting resistance mechanisms within specific patient-derived microenvironments, thereby facilitating the identification of optimal treatment populations and advancing oncology therapeutics toward precision medicine paradigms grounded in ex vivo functional validation [29].

### 5.4. Immunotherapy Evaluation

Organoids-on-a-chip platforms reconstruct functionally active tumor immune microenvironments through three-dimensional co-culture of patient-autologous or engineered immune effector cells with tumor organoids under dynamic perfusion [30]. The three-dimensional spatial architecture supports the simulation of immune cell infiltration into tumor parenchyma, while controlled fluidic shear forces and nutrient gradients sustain the long-term activation and effector functions of immune cells. These systems enable systematic evaluation of the on-target cytotoxic efficacy, off-target toxicity, and resistance evolution of immunotherapeutic strategies including immune checkpoint modulators, cellular therapies, and bispecific antibodies, providing high-fidelity in vitro models for optimizing combination regimens, elucidating immune escape mechanisms, and predicting patient response tendencies.

### 5.5. Disease Molecular Mechanisms

In studying disease molecular mechanisms, organoids-on-a-chip platforms have emerged as powerful tools for modeling tissue-specific pathophysiology and dissecting molecular pathways in a controlled microenvironment. For instance, intestinal organoid chips have been used to elucidate how mechanical forces and biochemical gradients synergistically regulate Wnt/β-catenin signaling during colorectal cancer progression, while brain organoid chips enable real-time tracking of tau protein propagation and synaptic dysfunction in Alzheimer’s disease models. Liver-on-chip systems incorporating patient-derived hepatocytes have revealed metabolic reprogramming pathways in non-alcoholic steatohepatitis, including altered lipid metabolism and insulin resistance signaling. Patient-derived tumor organoids-on-a-chip faithfully retain the mutational landscape and stromal architecture of original tissues, enabling recapitulation of molecular events during the metastatic cascade, including epithelial-to-mesenchymal transition and invasion dynamics. Beyond mechanistic studies, organoids-on-a-chip leverage their biomimetic microenvironment to accelerate disease model construction and molecular target validation in drug screening. By integrating high-throughput microfluidics with automated imaging, these platforms enable parallel testing of compound libraries and real-time monitoring of molecular responses at single-organoid resolution, facilitating identification of drug-resistant subclones and their associated molecular fingerprints. At the personalized medicine level, the tumor-stromal genetic signatures and drug response profiles preserved on chip can effectively inform patient-specific therapeutic outcomes, and organoid models for pancreatic, colorectal, and hepatic malignancies have been successfully applied to molecular therapeutic screening, guiding treatment selection based on individual mutational profiles. Overall, organoids-on-a-chip technology is emerging as a versatile platform that bridges molecular pathway elucidation, target discovery, and clinical efficacy prediction [31].

## 6. Future Research Prospects

Organoids-on-a-chip are not conceived as wholesale replacements for animal models, but rather as strategically valuable, complementary evaluation tools that reconstruct the complete in vivo trajectory of a drug candidate from absorption and distribution, through metabolism and target engagement, to the onset of toxicological responses. Their true value does not reside in the mere morphological mimicry of miniature organs; instead, it lies in the dynamic, physiologically relevant integration of three critical elements: (i) human cellular diversity, often derived from patient-specific or genetically engineered cell sources; (ii) precise control over the biochemical and biophysical microenvironment, including fluid shear stress, oxygen gradients, and nutrient supply; and (iii) multi-organ serial exposure, where interconnected tissue chambers replicate the sequential passage of drugs and their metabolites across different organ barriers. This integrative capability directly addresses the two fundamental limitations of conventional preclinical models. Namely, the static, closed culture conditions that fail to mimic circulatory dynamics and the persistent interspecies disparities that confound extrapolation of human responses. By incorporating continuous microperfusion, real-time biosensing, and co-culture architectures, enabling early de-risking of candidates and more informed prioritization before committing to expensive animal studies. In this context, they function not as substitutes but as powerful filters and bridge systems, enhancing the translational relevance of preclinical data while reducing reliance on animal testing, thereby accelerating the development of safer and more efficacious therapeutics.

Despite significant advances, the broad deployment of organoids-on-a-chip technology across the entire drug development pipeline remains constrained by several critical challenges. First, variability in organoid differentiation batches, chip fabrication tolerances, and fluidic parameter control across laboratories limits experimental reproducibility; establishing reference cell lines, standardized reference compound libraries, and open-access data standards has become a foundational imperative for the field. Second, the generally low culture throughput of current platforms cannot meet the demands of large-scale compound screening in early-stage drug discovery; enhancing system automation and parallel processing capabilities represents a key developmental priority to overcome this bottleneck. Third, with the exception of a few preliminarily accepted models, most organoids-on-a-chip systems have not yet received formal Context of Use designation from regulatory agencies; each specific application scenario requires prospective, blinded validation against human clinical data to achieve regulatory acceptance. Furthermore, the absence of functional vascular perfusion networks in most current models restricts long-term culture stability and the ability to resolve dynamic biological processes such as immune cell homing; developing perfusable vascularized systems and integrated immune co-culture models is essential to advancing physiological relevance. Realizing the translational potential of organoids-on-a-chip requires a paradigm shift in the field from structural simulation to predictive performance validation. Specifically, each chip model should be purpose-built and validated for a well-defined application context, with validation endpoints extending beyond transcriptomic similarity to prospective, blinded comparison against human clinical data. At the materials level, the development of new biomaterials, including stiffness-programmable hydrogels and tissue-specific decellularized extracellular matrix composites, continues to enhance the physiological fidelity of these models. At the systems integration level, advances in modular connectivity strategies have substantially lowered the technical barriers to multi-organoids integration, enabling researchers to flexibly assemble customized ADME testing platforms in a plug-and-play manner. Looking ahead, liver organoids-on-a-chip and heart organoids-on-a-chip systems are poised to become routine supplementary data modules in Investigational New Drug applications. Tumor organoids-on-a-chip platforms will be employed in companion diagnostics for functional drug sensitivity profiling; and multi-organoids-on-a-chip will primarily address complex toxicity assessment scenarios that are poorly predicted by conventional animal models, thereby furnishing drug development decisions with more human-specific in vitro evidentiary support.

Finally, artificial intelligence is actively reshaping organoids-on-a-chip research through direct integration with experimental and analytical workflows. In image analysis, deep learning algorithms enable automated segmentation, classification, and tracking of organoid morphologies, supporting high-content phenotypic screening and real-time monitoring of cellular dynamics. These capabilities facilitate automated quality control by detecting structural abnormalities, assessing organoid viability, and standardizing culture conditions across replicate experiments. AI-driven integration of multi-omics data—including transcriptomics, proteomics, and metabolomics—has demonstrated practical utility in biomarker discovery, optimization of combination drug therapies, and prediction of individual treatment responses. Additionally, the combination of spatial transcriptomics with single-cell profiling enables quantitative reconstruction of cellular state transitions during disease progression, offering measurable insights into drug–target interactions and pathway reprogramming. In toxicity assessment, machine learning models trained on morphological and molecular features provide predictive tools for early identification of adverse drug reactions. Through intelligent analysis of molecular imaging and mass spectrometry data, AI enables multi-level quantitative characterization spanning from cellular phenotypes to molecular events. The practical deployment of AI in organoids-on-a-chip systems has improved the accuracy of drug response evaluation and reduced experimental variability, thereby strengthening the transition from conventional animal-based testing to human-relevant platforms that support personalized therapeutic strategies.

## Figures and Tables

**Table 1:** Materials used in chip fabrication and their respective advantages and disadvantages.

Material Category	Representative Materials	Advantages	Disadvantages
Elastomers	PDMS	Transparent, elastic, biocompatible, easy to process, suitable for rapid prototyping	Strongly hydrophobic, adsorbs small molecules, interferes with drug testing assays
Thermoplastics	PMMA, PC	Mass producible, good mechanical strength, transparent, low absorption of small molecules	Rigid, lacking elasticity, poor gas permeability
Hydrogels	Collagen, gelatin, alginate, PEG	Biomimetic microenvironment, mimics native ECM, high biocompatibility	Batch-to-batch variability, insufficient mechanical strength, degrades over long-term culture
Novel Functional Polymers	Decellularized ECM, self-assembling peptides	High bioactivity, retains native ECM components and structure	Complex preparation, limited sources, high cost

## Data Availability

The data that support the findings of this study are available from the corresponding author upon reasonable request.
